# Genome-wide computational identification of functional RNA elements in *Trypanosoma brucei*

**DOI:** 10.1186/1471-2164-10-355

**Published:** 2009-08-04

**Authors:** Yuan Mao, Hamed Shateri Najafabadi, Reza Salavati

**Affiliations:** 1 Institute of Parasitology, McGill University, 21,111 Lakeshore Road, Ste. Anne de Bellevue, Montreal, Quebec H9X3V9, Canada; 2 McGill Centre for Bioinformatics, McGill University, Duff Medical Building, 3775 University Street, Montreal, Quebec H3A2B4, Canada; 3 Department of Biochemistry, McGill University, McIntyre Medical Building, 3655 Promenade Sir William Osler, Montreal, Quebec H3G1Y6, Canada

## Abstract

**Background:**

Post-transcriptional regulation of gene expression is the dominant regulatory mechanism in trypanosomatids as their mRNAs are transcribed from polycistronic units. A few *cis*-acting RNA elements in 3'-untranslated regions of mRNAs have been identified in trypanosomatids, which affect the mRNA stability or translation rate in different life stages of these parasites. Other functional RNAs (fRNAs) also play essential roles in these organisms. However, there has been no genome-wide analysis for identification of fRNAs in trypanosomatids.

**Results:**

Functional RNAs, including non-coding RNAs (ncRNAs) and *cis*-acting RNA elements involved in post-transcriptional gene regulation, were predicted based on two independent computational analyses of the genome of *Trypanosoma brucei*. In the first analysis, the predicted candidate ncRNAs were identified based on conservation with the related trypanosomatid *Leishmania braziliensis*. This prediction had a substantially low estimated false discovery rate, and a considerable number of the predicted ncRNAs represented novel classes with unknown functions. In the second analysis, we identified a number of function-specific regulatory motifs, based on which we devised a classifier that can be used for homology-independent function prediction in *T. brucei*.

**Conclusion:**

This first genome-wide analysis of fRNAs in trypanosomatids restricts the search space of experimental approaches and, thus, can significantly expedite the process of characterization of these elements. Our classifier for function prediction based on *cis*-acting regulatory elements can also, in combination with other methods, provide the means for homology-independent annotation of trypanosomatid genomes.

## Background

RNA elements that are functional at RNA level, i.e., functional RNAs (fRNAs), are becoming to be appreciated more and more as their diverse structural, regulatory and catalytic roles are revealed [1,2]. Several classes of fRNAs have been identified, including different types of non-coding RNAs (ncRNAs) such as tRNAs, rRNAs, microRNAs (miRNAs), Telomerase RNA, RPR1 (the RNA component of nuclear RNase P), small nuclear RNAs (snRNAs) and small nucleolar RNAs (snoRNAs). The *cis*-regulatory elements in the 5'- and 3'-untranslated regions (UTRs) of mRNAs constitute another class of fRNAs that are mostly involved in post-transcriptional regulation of gene expression (see [3,4]). Recent developments in computational tools for prediction of fRNAs have shown a widespread set of RNA elements that are specifically involved in post-transcriptional regulatory processes [5]. Although crucial in many different species, post-transcriptional regulation is especially the major mechanism for regulation of gene expression in a group of unicellular parasites called trypanosomatids.

Trypanosomatids, including *Trypanosoma brucei*, *T. cruzi *and different *Leishmania *species, are the causative agents of serious human as well as animal diseases, with a very high incidence and mortality rate if untreated. Genes in trypanosomatids are transcribed as polycistronic mRNAs [6] that are further processed via trans-splicing [7]. Regulation of gene expression which occurs mostly during or after splicing involves several *cis*-acting fRNA elements, such as U-rich elements (UREs), short interspersed degenerated retroposons (SIDERs), etc. [3,4]. These elements mostly regulate either the stability or translation rate of mRNAs via interaction with different *trans*-acting proteins, many of which are unknown. It has also been proposed recently that miRNAs may play a role in posttranscriptional gene regulation in *T. brucei *[8], although no experimental substantiation has been found.

Experimental identification of *cis*-acting fRNA elements is an exhausting task that requires extensive functional assays with several strains carrying deletion/substitution mutants of a likely regulatory sequence. The situation is not better for ncRNAs, as it is not clear in which region(s) in the genome they should be searched for and for what particular function the screening experiment should be designed (as opposed to *cis*-acting fRNA elements that occur adjacent to coding sequences and affect gene expression). Although computational identification of fRNAs from genome sequences can be an alternative, it is not yet as robust as identification of protein-coding RNAs, due to the lack of strong conserved signals in their sequences [9]. Here, we present a computational examination of the genomes of *T. brucei *and *L. braziliensis *in order to identify a set of conserved ncRNAs that, based on computational and statistical analysis, are highly reliable. We show that our methodology is able to find a large number of known as well as novel potential ncRNAs. We further examine our candidate ncRNAs for the presence of potential pre-miRNAs, and show that the existence of miRNA genes that are conserved between *T. brucei *and *L. braziliensis *is highly unlikely. We also use a different method for homology-independent identification of short regulatory RNA motifs in 5' and 3' UTRs of *T. brucei *genes. These motifs complement our predicted ncRNAs by providing a set of the most functionally important regions of potential *cis*-regulatory fRNA elements. In addition to offering new insights about the regulatory mechanisms of protein expression in *T. brucei*, these regulatory motifs can be used for prediction of gene function.

## Results and Discussion

### Identification of conserved ncRNAs in *T. brucei*

We compared the genome sequences of *T. brucei *and *L. braziliensis *in order to identify conserved genomic regions. *L. braziliensis *is the only trypanosomatid other than *T. brucei *with available genome sequence in which the putative components of RNAi machinery have been identified [10]. Thus, its comparison with *T. brucei *provides the possibility of detecting conserved ncRNAs involved in or processed by this machinery. We used a binomial-based model [11] to assess the conservation across *T. brucei *genome in comparison to the genome sequence of *L. braziliensis*. Using this model, we found that about 18% of the *T. brucei *genome shows conservation degrees above the median that would be expected from a random distribution. These regions, in addition to being enriched for functional elements, have allegedly the highest-quality alignments compared to the alignments that correspond to less conserved regions. This conserved subset of the *T. brucei *genome consisted of about 5.26 Mbp of protein-coding sequences and 887 kbp of non-coding sequences. We used QRNA [12] to identify parts of these conserved genomic regions that showed patterns of conserved structural RNA elements. About 37.2 kbp of the non-coding conserved genomic regions obtained RNA scores above zero, using QRNA. For the protein-coding conserved regions, this number was about 16.8 kbp, indicating a false positive rate of about 0.3%. Assuming this false positive rate, we would expect about 2.8 kbp of false positives among non-coding genomic regions and, hence, a precision of about 92.3% (precision was defined as TP/(TP+FP), where TP and FP stand for the number of true positives and false positives among non-coding genomic regions, respectively).

It should be noted that the estimated false positive rate from coding sequences would not be applicable to non-coding sequences if we had included scores other than the RNA score from QRNA, such as the COD and OTH scores (COD and OTH scores express the likelihood of being a coding sequence and a non-RNA, non-coding sequence, respectively). However, the behavior of QRNA may still be different between coding regions and non-RNA, non-coding genomic regions as coding sequence evolves in a very specific way. Furthermore, RNA structure in coding sequence may be specifically selected against. We have thus used a different, more conservative method for estimating the false positive rate of our ncRNA predictions, which is explained in the section "Identification of highly significant candidate ncRNAs".

About 5.2 kbp of our found candidate fRNAs overlapped with already annotated rRNA, snRNA, and tRNA genes, indicating the capability of our approach in finding non-coding RNAs. The sensitivity of this approach, i.e., TP/(TP+FN) where FN indicates the number of false negatives, showed considerable differences among different classes of structural RNAs. For example, 30 of our predicated candidates overlapped one of the 65 known tRNAs, equal to about 50% sensitivity for detection of tRNAs. On the other hand, only 21 candidates overlapped one of the 106 known rRNA genes, indicating a lower sensitivity for rRNA detection. This is while we detected none of the 353 known small nucleolar RNAs (snoRNAs). This may indicate the lack of conservation of snoRNA structure between *T. brucei *and L. *braziliensis*.

**Figure 1:**
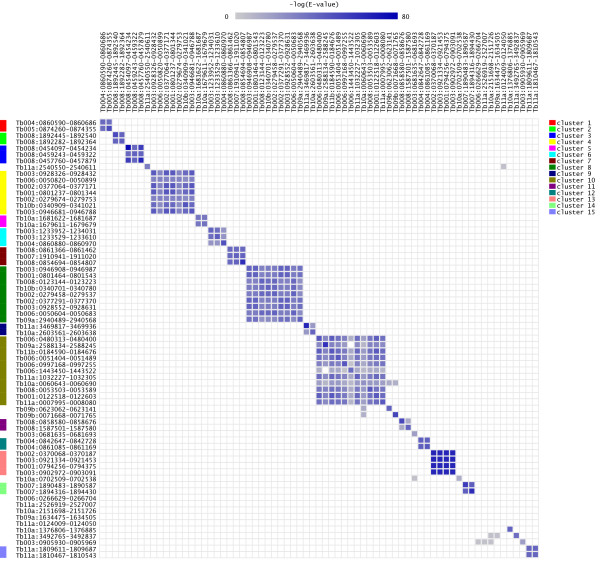
**Homology table for the predicted ncRNAs**. Many candidate ncRNAs can be grouped into several homology clusters, here shown by color labels (clusters 1–15). In this figure, only ncRNAs are shown for which there is at least one other predicted ncRNA with homology E-value < 0.0025 and alignment coverage > 50%. The color of each square reflects the BLAST E-value with the sequence in the corresponding row as the query.

A complete list of all found ncRNA candidates along with their associated information can be found in Additional File 1. Many of these candidates can be grouped into several homology clusters, as shown in Figure 1. When several homologous sequences are independently predicted to be ncRNAs, the predictions can be considered highly reliable. Sequences within clusters 1, 2, 6, 7, 9, 11, 12 and 14 either overlap with or are homologous to known tRNAs. Similarly, sequences within clusters 3, 4, 8 and 13 seem to represent rRNAs. However, clusters 5, 10 and 15 do not correspond to any known ncRNAs and, thus, may represent novel ncRNA classes with unknown functions. Cluster 10 is of particular significance due to its large size, indicating that the elements of this class may be present at a high frequency in the genome.

### Investigating the presence of conserved miRNA genes

Based on a computational analysis of *T. brucei *genome, it has been recently proposed that trypanosomatids may use miRNAs in order to regulate the levels of particular mRNAs [8]. However, this report is not consistent with our current knowledge of miRNA origin [13,14]: regulation via miRNA seems to have emerged in a completely different branch of life, although its convergent evolution in several branches is not impossible. Hence, we decided to investigate the presence of putative miRNA precursors among our predicted ncRNAs through a relatively simple, yet specific approach that considers a few structural and thermodynamic criteria for identification of pre-miRNA sequences (see Methods section). Using 250 pre-miRNAs that, as control sequences, were randomly selected from 24 different organisms (Additional File 2), it can be estimated that the sensitivity of pre-miRNA prediction using our criteria is about 32.4% ± 2.1%. Also, using a set of 30770 randomly selected sequences from *T. brucei *genome, the specificity of this method can be estimated at about 99.1% ± 0.3%.

After removing low-complexity regions (LCRs, see the Methods section), only five of the predicted ncRNA sequences met our criteria for structure and free folding energy (Additional File 3). However, these rare sequences are mostly consisted of dinucleotide repeats (particularly AU repeats), and can be accounted for false positives of our method. Based on our analysis, it is highly unlikely to expect any conserved miRNA genes in *T. brucei*. It should be mentioned that a large number of the previously predicted *T. brucei *miRNAs [8] were potentially targeting variant surface glycoproteins (VSGs), which are absent in *L. braziliensis*. However, other predicted miRNAs were targeting conserved complexes such as 20S proteasome, and thus would be expected to be found in this study if they were conserved. Although this does not exclusively reject the presence of miRNAs in *T. brucei *genome, suggests that a reexamination of this genome for the presence of such elements is required.

### Identification of highly significant candidate ncRNAs

In order to select a highly significant subset from our set of candidate conserved ncRNAs, we filtered out the candidates whose QRNA scores were not significantly higher than expected from a random distribution. The random distribution for each candidate ncRNA was obtained by computing the QRNA scores of 1000 randomly scrambled *T. brucei*-*L. braziliensis *alignments, as described in the Methods section. A candidate ncRNA was rejected if it was outscored by more than three randomized versions (i.e., p ≤ 0.003; this p-value threshold was selected so as the expected number of false positives would be less than one). This filtering procedure resulted in 117 highly significant novel putative ncRNAs (Additional File 4 and the first 117 candidates in Additional File 1), of which 53 neither overlapped nor were homologous to any annotated features of *T. brucei *genome and, hence, may represent completely novel ncRNAs (Table 1). All 117 candidates that did not overlap with a coding sequence had the highest score for the RNA model and not the COD and OTH models, although they were initially selected only based on their RNA scores and irrespective of their COD and OTH scores.

**Table 1:** Classification of predicted ncRNAs in *T. brucei *genome

Classification	Within 100 bp of a non-overlapping coding sequence**	Within/flanking ncRNA cluster (no. of ncRNAs >2)***	Within a strand switch region***	Elsewhere	Total
Overlap CDS	0	0	0	36	**36**
Overlap pseudogene	0	0	0	1	**1**
Overlap unlikely proteins	0	0	0	0	**0**
Homologous to rRNA*,**	0	4	2	1	**5**
Homologous to tRNA*	1	0	0	0	**1**
Overlap known ncRNA**	0	25	7	1	**26**
Overlap Ingi/RIME repeat	0	0	0	0	**0**
Unclassified	8	1	1	43	**53**
**Total**	**9**	**26**	**8**	**81**	**117**

Candidate ncRNAs are classified based on either homology with known ncRNAs or overlap with known genomic features. Candidate ncRNAs within each class are further divided into subgroups based on their location relative to known genomic features.

* Each candidate may contain several closely located single ncRNAs, some of which may have already been annotated on the current release of the *T. brucei *genome. However, at least one ncRNA within each sequence is unannotated, for which a known homolog is found. These unannotated ncRNAs represent novel instances of their classes.

** These categories may overlap.

*** These categories may overlap.

The calculated p-value provides another measure, though more conservative, for estimating the precision of our method. For example, a p-value ≤ 0.001 is equal to about 0.887 kbp of false positives (out of 887 kbp of the non-coding conserved genomic regions), assuming that most of the non-coding genome is consisted of non-RNA random sequences. This is while more than 5.7 kbp of our candidates (the top 79 candidates in Additional File 1) were significant at this level, indicating a precision of about 85% at this level of significance.

These novel ncRNAs did not show any statistically significant enrichment in particular genomic positions such as regions with clustered ncRNAs, strand switch regions (regions where the coding strand changes) or regions adjacent to coding sequences (significance was defined as p-value < 0.05 in a genomic position permutation test), indicating a relatively uniform distribution on the genome. Nonetheless, a crude guess can be made for biological function of some candidates based on their positions. For example, eight unclassified candidate elements occur in the vicinity of a coding sequence. These elements may represent regulatory structures at 5' or 3' UTRs of coding sequences, involved in post-transcriptional regulation of gene expression. Also, one unclassified candidate fRNA was found to occur in a strand switch region. As transcription of polycistronic mRNAs start from strand switch regions, this fRNA may represent an element in the 5' end of the resultant transcript, and may be involved in its localization, posttranscriptional processing or regulation.

Expectedly, none of the previously characterized *cis*-regulatory RNA elements of *T. brucei *were found among our set of candidate structural RNA elements. This is not surprising since the known regulatory RNA elements of *T. brucei *are not conserved in *Leishmania *species [3,4]. Furthermore, many of these elements are known via their sequence, not their structure. We specifically discuss the computational identification of *cis*-regulatory RNA elements in *T. brucei *in the next section.

### Finding informative function-specific regulatory elements

**Figure 2:**
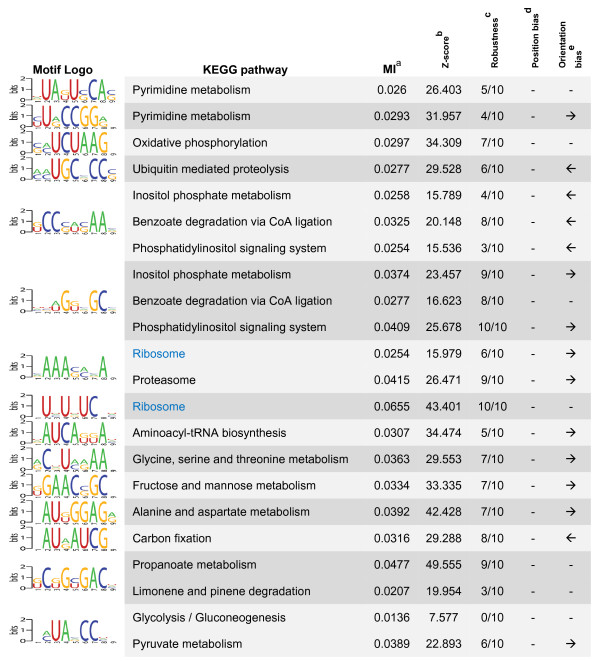
**Function-specific motifs in 5' UTRs of *T. brucei *genes**. The functions in which each motif is significantly overrepresented or underrepresented are indicated in the second column using black and blue text colors, respectively. Column headings: (a) Mutual information value; (b) Z-score associated with the MI value; (c) Robustness, obtained from ten jack-knife trials of randomly removing one-third of the genes and reassessing the statistical significance of the resulting MI values; (d) Position bias indicator – "Y" if a position bias is observed; (e) Orientation bias, indicating the orientation of the motif with respect to its associated coding sequence.

**Figure 3:**
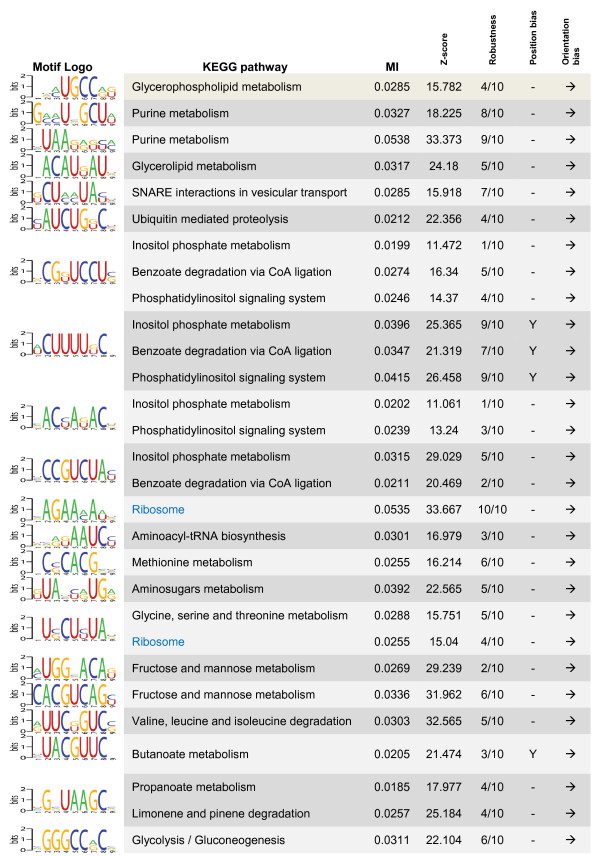
**Function-specific motifs in 3' UTRs of *T. brucei *genes**. The functions in which each motif is significantly overrepresented or underrepresented are indicated in the second column using black and blue text colors, respectively. Column headings are the same as in Figure 2.

We used a homology-independent approach to investigate the presence of function-specific motifs in 5' and 3' UTRs of *T. brucei *genes, using a recently developed algorithm, named FIRE [15]. It has been shown that FIRE is able to identify many known and novel regulatory elements, with a near-zero false positive discovery rate, in upstream and downstream of genes that are clustered according to their expression patterns. Here, we used FIRE to find 'function-specific' regulatory elements in 5' and 3' UTRs of *T. brucei *genes: genes with similar functions are usually co-regulated [16], indicating that they should have similar *cis*-regulatory elements. Thus, clustering genes according to their functions can be used as a surrogate of clustering them according to their expression patterns. This approach is particularly useful for organisms in which gene regulation occurs mostly at post-transcriptional levels, such as trypanosomatids (transcript profiling studies cannot identify the dynamics of protein expression in such organisms; see [17]). We were able to identify 15 function-specific motifs in 5' UTRs of *T. brucei *genes and 21 function-specific motifs in their 3' UTRs (Figures 2 and 3 and Additional File 5). Based on the results of running FIRE on 10 permuted sets of gene-function assignments, we can estimate an expected precision of 75.3% for discovering function-specific 5' UTR motifs and 84.8% for 3' UTR motifs.

Most of the motifs that are found by FIRE have orientation bias, i.e., mostly occur at a particular orientation with respect to the coding sequence. This property is expected from RNA motifs. Furthermore, two of the motifs that were predicted in 3' UTRs have position bias, which means that they prefer to be at a particular distance from the stop codon of the upstream coding sequence. This property has also been observed for many regulatory motifs in different organisms [15,16], and further increases the possibility that the predicted motif has a biological role.

Our predicted function-specific motifs overlap with a number of experimentally found regulatory sequences in *T. brucei*, mostly identified by deleting different parts of UTRs and evaluating the effects of these deletions on regulation of a reporter gene: It has been shown that the 3' UTR of glycosomal phosphoglycerate kinase PGKC can cause bloodstream form-specific gene expression [18,19]. We found that this regulatory sequence contained six of our predicted 3' UTR motifs (p < 1 × 10^-5^), most notably the glycolysis-specific motif VGGGCCRCV (degenerate positions are shown using IUPAC nomenclature of mixed bases [20]). Interestingly, the 5' UTR of the same gene, which has been shown to affect splicing in procyclic stage [21,22], also contains two copies of the 5' UTR motif UHUDUCNH. As another example, the 3' UTR of fructose bisphosphate aldolase contains an instance of the fructose metabolism-specific motif MUGGVACAK. This untranslated region has also been reported to be able to cause regulated expression of genes in *T. brucei *[18].

It should be noted that our approach is only able to identify function-specific short RNA motifs, not motifs that are involved in regulation of expression in a rather genome-wide scope, or in a gene-specific manner. Thus, it is not surprising to see that some of the previously identified regulatory elements, such as the widely used U-rich elements [4] are not among our motifs. Structural RNA elements also cannot be identified using FIRE; nonetheless, some of our found short motifs may represent the most functionally important regions of RNA structural elements.

### Function prediction using regulatory RNA motifs

**Figure 4:**
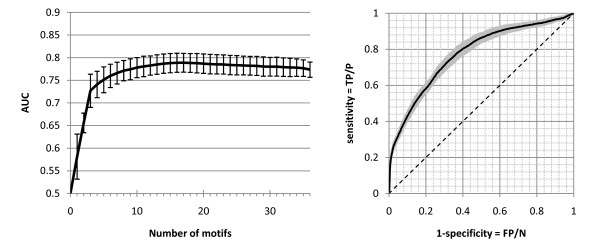
**Function prediction using regulatory motifs in *T. brucei***. This figure shows inositol phosphate metabolism (KEGG:tbr00562) as an example. **(A) **The performance of our naïve Bayesian network using different numbers of motifs for prediction of inositol phosphate metabolism genes. We used a two-fold cross-validation for assessing the prediction power, where half of the dataset was used for training and the other half for validation. Cross-validation was repeated 100 times for each number of motifs, and each time the AUC (area under the curve) of the ROC curve was measured as the prediction power. Standard deviation of AUC is shown by the error bars. **(B) **The ROC curve for prediction of inositol phosphate metabolism genes using all 36 predicted motifs. Standard deviation of sensitivity is shown by the grey shaded region. The diagonal line shows the performance that would be expected if our naïve Bayesian network was not able to predict inositol phosphate metabolism genes. This classifier has a very high specificity (~99%) at sensitivities of up to 20% for this pathway.

We devised a naïve Bayesian network that based on the pattern of presence and absence of motifs in 5' UTRs and 3' UTRs can predict whether a gene belongs to a particular pathway (see Methods section). For many pathways, this naïve Bayesian network can be used to classify *T. brucei *genes with acceptable reliability (see Figure 4 for an example). As it is shown in Figure 4A, only a few motifs are needed to reach the maximum possible prediction power. However, adding more motifs to this classifier does not reduce the prediction power, which simplifies the design of effective naïve Bayesian networks. We expect that by combining this method with other function prediction methods, we will be able to expand the functional annotations of *T. brucei *genes extensively. A complete assessment of function prediction in *T. brucei *using our method can be found in Additional File 6.

## Conclusion

The ncRNAs predicted in this study can provide candidates for experiments that are focused on understanding the functional RNA repertoire of trypanosomatids. The most interesting candidates are perhaps those that do not have characterized homologs, as they most probably represent novel ncRNA classes in *T. brucei*. Unraveling the function of these ncRNAs will help us to understand the biology of these parasites more clearly. However, it should be noted that our set of predicted ncRNAs is far from complete, as we only considered two genomes in this study. Considering a larger number of trypanosomatid genomes may reveal other ncRNAs and provide a more thorough view of the non-coding functional transcriptome of these organisms.

Prediction of gene functions based on our set of function-specific short motifs can also provide a very useful alternative to homology-based annotation methods, especially that a huge number of trypanosomatid genes are not conserved in other organisms. We anticipate that combining this method with other established systems-based function prediction approaches provides a robust method that can be applied to many genomes.

## Methods

### Identification of conserved genomic regions

Conserved genomic regions were identified using a binomial-based model [11]. Briefly, after masking LCRs using mreps, the two genomes of *T. brucei *and *L. braziliensis *were aligned using BlastZ [23]; for each window of 25 nucleotides, *N*, the number of conserved nucleotides, was determined; the probability of observing *N *conserved nucleotides out of 25 nucleotides was calculated under the null hypothesis of neutral substitution and based on binomial distribution of mutations; and regions showing evidence of negative selection were chosen. Finally, conserved regions that were less than 25 nucleotides apart were connected to each other as a single region (see [11] for detailed description of the method).

### Identification of conserved ncRNAs

We used QRNA [12] to identify parts of the conserved genomic regions (see above) that showed patterns of conserved structural RNA elements. Long sequences were broken into smaller overlapping fragments of 80 nucleotides, each of which having 40 nucleotides overlap with its adjacent fragments. Overlapping sequences with RNA scores higher than zero were merged again, QRNA scores were recalculated, and those with final positive RNA scores were selected as putative fRNAs. False positive rate was calculated as the fraction of conserved coding sequences that were classified as fRNA. LCRs were determined using a combination of 'mreps' and 'mdust' from TIGR, marked in Additional File 1 by lowercase letters. Significance of each candidate was assessed by comparing its QRNA score to a distribution obtained by randomizing *T. brucei*-*L. brazilensis *alignments: the alignment of each ncRNA candidate was considered separately, and columns with similar conservation patterns were shuffled randomly (i.e., a column containing a gap was swapped only with another gap-containing column, mismatch with mismatch, and match with match [24]). The fraction of random alignments outscoring the original alignment was considered as the p-value (Additional File 1, column H).

### Examining the candidate ncRNAs for the presence of potential pre-miRNAs

We used a set of simple, yet powerful criteria for detection of potential miRNA precursors among our candidate fRNAs. A nucleotide sequence of length 80 nt was considered a potential pre-miRNA if it could be folded into a structure with **(i) **a single stem-loop **(ii) **whose free folding energy was <= -25 kcal/mol [25], **(iii) **which contained an at least 9 bp-long continuous paired region with no internal loops or bulges, **(iv) **and contained no unpaired internal segment (internal loop or bulge) longer than 3 nucleotides. These criteria, while selected empirically to optimize for specificity and sensitivity, are in agreement with previous studies on pre-miRNA structure [26]. We used RNAfold from Vienna RNA package for folding the sequences.

The sensitivity and specificity of these criteria were tested on a set of 250 pre-miRNAs from 24 different organisms (Additional File 2) and a set of 30770 randomly selected sequences from *T. brucei *genome (random sequences matching the selected criteria were considered false positives, based on which the specificity was estimated). To estimate the standard deviations of sensitivity and specificity, we performed 10 jack-knife trials in each of which one third of all sequences were randomly removed and the performance was reevaluated on the remaining two thirds. Then, all *T. brucei *genomic sequences of length 80 nt which overlapped with at least one nucleotide of one of the predicted ncRNAs, as well as the reverse complements of such genomic sequences, were examined using the above criteria for presence of pre-miRNAs.

### Finding informative regulatory elements in 5' and 3' UTRs

A recently developed algorithm, named FIRE, is able to identify DNA and RNA motifs that are unevenly distributed among different clusters of sequences, i.e., are overrepresented in some clusters while underrepresented in some others [15]. Here, we used FIRE to identify motifs that are unevenly distributed among different functions. Functional annotations of *T. brucei *genes were retrieved from KEGG pathway database [27]. For each pathway, we grouped the genes into two clusters based on whether they were involved in that pathway or not. Then we used FIRE to find 5' UTR or 3' UTR motifs that showed significant overrepresentation or underrepresentation in either of the two clusters. The sequences of mature 5' and 3' UTRs were isolated from *T. brucei *genome based on splicing site predictions reported previously [7]. The resulting motifs from different functions were collected, and duplicate motifs were removed.

The same procedure was repeated for 10 sets of randomly shuffled gene-function assignments, and the average number of motifs reported by FIRE was used as an estimate of the expected number of false positives. The expected precision was consequently calculated as E(*Precision*) = E(*TP*)/*P *= [*P*-E(*FP*)]/*P*. Here, E(*X*) denotes the expected value of *X*, *TP *stands for true positive, *P *stands for positives (motifs detected by FIRE from actual dataset), and *FP *indicates false positives (motifs detected by FIRE from shuffled datasets).

It should be noted that KEGG annotations may not correspond to the precise function of the genes, since KEGG uses an automated pipeline for assigning the genes to template pathways based on homology with known proteins. However, we expect that the relationships of the genes are conserved through this procedure, e.g., if two genes are assigned to the same pathway in KEGG, they most probably have very closely related functions, even if the exact assigned functions in KEGG are not correct.

### Function prediction using regulatory motifs

We used a naïve Bayesian network to predict gene-function assignments based on the predicted regulatory motifs in 5' and 3' UTRs. Naïve Bayesian networks assume that the properties based on which they classify the objects are independent. Thus, the likelihood that gene *g *belongs to cluster *α *given a set of known motifs is calculated as:

Here, *M *is the set of motifs that are used for classification of *g*, and *F*^*M *^= {*f*_1_, *f*_2_, ..., *f*_|M|_} where *f*_*i *_is {1} if the *i*th motif is present in gene *g*, and {0} otherwise. *L*(*g *∈ α | *F*^*M*^) is the likelihood that *g *belongs to *α *given *F*^*M*^, and *L*(*g *∈ α | *f*_*i*_) represents the likelihood that *g *belongs to *α *given the status of the *i*th motif in *g*, i.e., *f*_*i*_. For more information about the calculation of conditional likelihoods and implementation of naïve Bayesian networks, refer to [28].

*M *is chosen in a way to maximize the prediction power. Briefly, motifs are selected iteratively, starting from the one that can best distinguish between *α *and *α*'. Then, at each iteration, all motifs are tested and the one whose addition to *M *results in the maximum prediction power is selected. Prediction power is assessed by the area under the ROC curve (Receiver Operating Characteristic or ROC curve plots sensitivity against false positive discovery rate). This procedure is repeated until *M *contains a predefined number of motifs. We removed paralogues prior to training and testing our naïve Bayesian network in order to avoid any biases towards duplicated UTRs.

## Authors' contributions

YM performed the analysis for identification of conserved ncRNAs. HSN performed the analysis for identification of short function-specific regulatory motifs, developed the algorithm for function prediction based on these elements, and wrote the manuscript. RS coordinated the study and provided intellectual input in all parts. All authors contributed in editing the manuscript.

## Supplementary Material

Additional file 1 **List of all conserved candidate fRNAs in *Trypanosoma brucei *genome**. Candidates with QRNA scores > 0.0 are included in this file. Explanation of the columns: **(A) **the nucleotide sequence of each candidate. Lower case letters indicate low complexity regions; **(B-E) **the chromosome where each candidate is located, its position on this chromosome, and its length, respectively; **(F-H) **QRNA score for the RNA model, Z-score, and the fraction of the shuffled variants scoring better than the candidate, respectively; **(I) **the annotated sequence closest to the left of the candidate (called L-neighbor), excluding unlikely proteins; **(J) **the exact description/name of the L-neighbor of the candidate; **(K) **the ID of the L-neighbor in the context of a working GeneDB link; **(L) **the distance between the candidate and its L-neighbor (red if within 100 nucleotides of a coding sequence, pink/purple if within 100 nucleotides of an ncRNA); **(M) **the strand on which the L-neighbor is found; **(N-R) **the same as I-M but for the nearest annotated sequence to the right of the candidate (called R-neighbor); **(S-U) **same as I-K for the annotated sequence(s) overlapping the candidate (called O-element); **(V) **the part of the candidate that overlaps its O-element sequence (left, right, middle or all). **(W) **the percentage of the candidate that overlaps its O-element. **(X) **strand on which the O-element is found; **(Y-AD) **the same as S-X, for the candidate's best homologous known ncRNA, with addition of the column AB (see below); **(AB) **the number of *T. brucei *ncRNA copies at different locations that are homologous to the candidate; **(AE) **the E-value of the best homologue; **(AF) **the homology cluster to which the candidate belongs (see Figure 1 of the main article); **(AG) **additional notes: whether the candidate is near unlikely proteins, known ncRNAs, strand-switch regions, etc. Click here for file

Additional file 2 **List of microRNA sequences that were used for assessing the sensitivity of our microRNA prediction method**. Accession numbers correspond to miRBase http://microrna.sanger.ac.uk/sequences/. Click here for file

Additional file 3 **miRNA-like predicted ncRNAs**. Candidate ncRNAs whose predicted secondary structures match our criteria for miRNA prediction are shown in this figure. It can be seen that their sequences are mostly consisted of AU repeats, rendering them unlikely candidates for being miRNA. Click here for file

Additional file 4 **The most significant ncRNA candidates in EMBL format**. We suggest viewing the files in this ZIP compressed package by Artemis http://www.sanger.ac.uk/Software/Artemis/. Open the EMBL file containing the sequence of each *T. brucei *chromosome (downloadable from http://www.ebi.ac.uk/genomes/eukaryota.html) by Artemis and then load the corresponding fRNA prediction file using "Read Entry..." option. This will add the predictions on the chromosome view with interactive capabilities. Click here for file

Additional file 5 **Function-specific regulatory motifs that were identified in 5' and 3' UTRs of *T. brucei***. Each row represents one motif, while each column stands for one function. Overrepresentation of a motif in a function is indicated by a yellow square, while underrepresentation is shown by blue. The probabilities of overrepresentation or underrepresentation were calculated based on hypergeometric distribution assumption and are shown here by the color gradient on log scale. Click here for file

Additional file 6 **Function prediction using regulatory motifs**. This ZIP compressed file should be unpacked before viewing its contents. The results for prediction of each pathway are presented in a separate folder, named after the accession number of that pathway in KEGG database. Each folder contains several files named "validation.*N*.out", each of which represents the results of a hundred times two-fold cross-validation for that pathway, using *N *predicted motifs. Each cross-validation is shown in one row; plotting each row against the number of false positives will result in the ROC curve associated with the corresponding cross-validation experiment. The trained network is stored in "total.out", in which motifs are ordered according to their prediction power. **L(α|MOTIF): **the likelihood of being in pathway α given that the indicated motif is present in the indicated location of an mRNA; **L(α|MOTIF)**: the likelihood of being in pathway α given that the indicated motif is NOT present in the indicated location of an mRNA. Click here for file
