# Supplementary material for: Genome-wide computational identification of functional RNA elements in Trypanosoma brucei
Source: BMC Genomics. 2009 Aug 4;10:355. doi: 10.1186/1471-2164-10-355 (PMC2907701; doi:10.1186/1471-2164-10-355)

Secondary structure: ΔG = -27.3kcal/mol

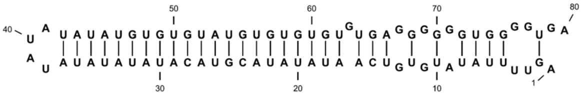

Secondary structure: ΔG = -26.3kcal/mol

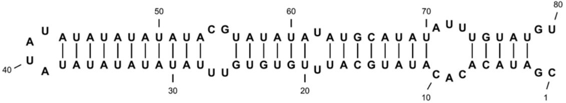

Secondary structure: ΔG = -26.0kcal/mol

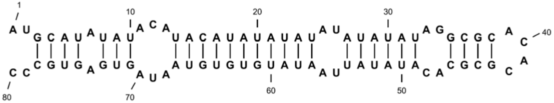

Secondary structure: ΔG = -27.0kcal/mol

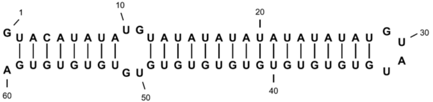

Secondary structure: ΔG = -26.5kcal/mol

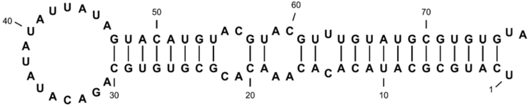

Supplement: Additional file 3 — miRNA-like predicted ncRNAs. Candidate ncRNAs whose predicted secondary structures match our criteria for miRNA prediction are shown in this figure. It can be seen that their sequences are mostly consisted of AU repeats, rendering them unlikely candidates for being miRNA. [file 1471-2164-10-355-S3.pdf]
