# Supplementary figures and images for: Genome-wide computational identification of functional RNA elements in Trypanosoma brucei
Source: BMC Genomics. 2009 Aug 4;10:355. doi: 10.1186/1471-2164-10-355 (PMC2907701; doi:10.1186/1471-2164-10-355)

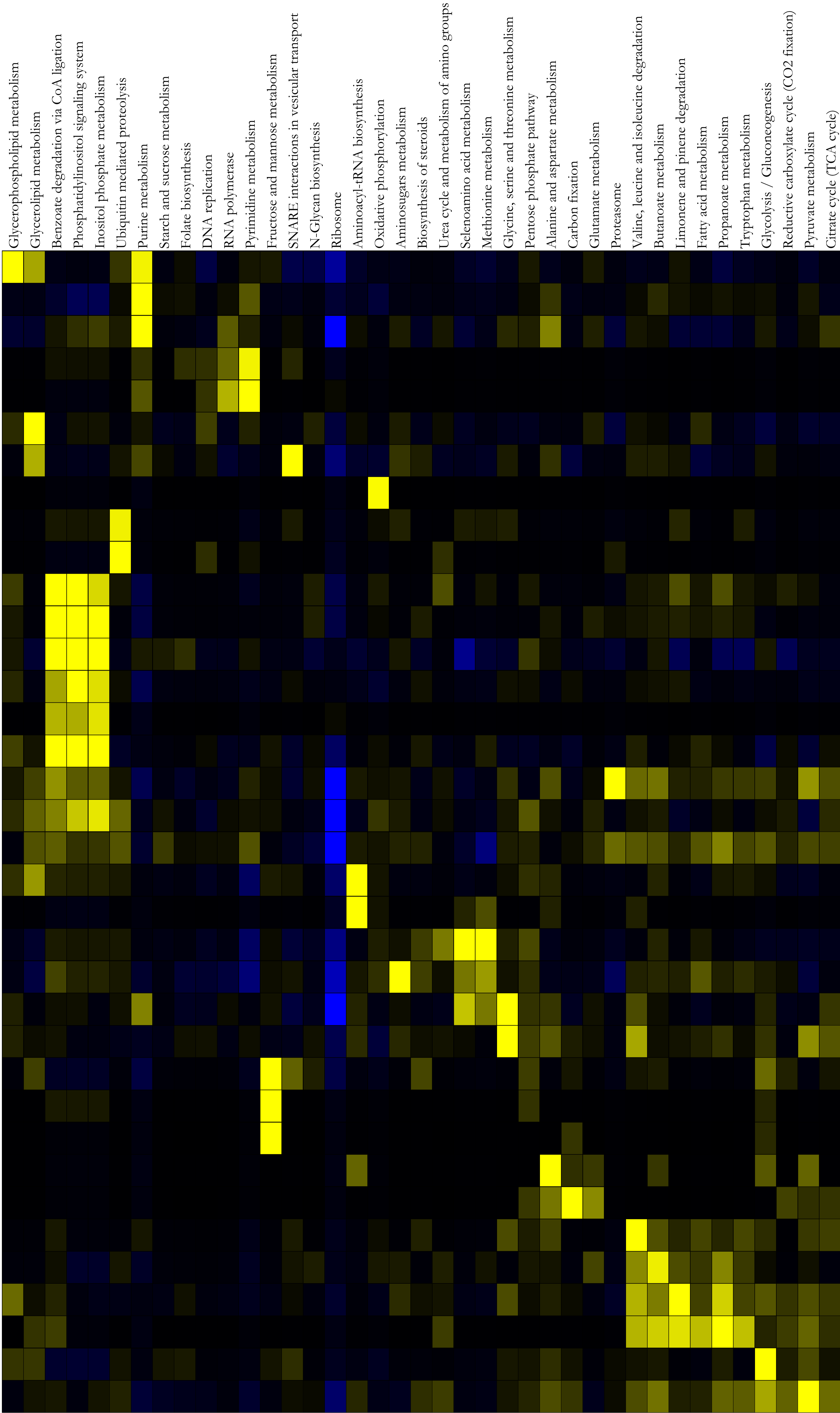

Supplement: Additional file 5 — Function-specific regulatory motifs that were identified in 5' and 3' UTRs of T. brucei. Each row represents one motif, while each column stands for one function. Overrepresentation of a motif in a function is indicated by a yellow square, while underrepresentation is shown by blue. The probabilities of overrepresentation or underrepresentation were calculated based on hypergeometric distribution assumption and are shown here by the color gradient on log scale. [file 1471-2164-10-355-S5.pdf]
